# Public acceptability and anticipated uptake of risk-stratified bowel cancer screening in the UK: An online survey

**DOI:** 10.1016/j.pmedr.2024.102927

**Published:** 2024-11-10

**Authors:** Lily C. Taylor, Rebecca A. Dennison, Juliet A. Usher-Smith

**Affiliations:** The Primary Care Unit, Department of Public Health and Primary Care, University of Cambridge, Cambridge, UK

**Keywords:** Acceptability, Risk stratification, Screening, Bowel cancer, Uptake

## Abstract

**Objective:**

This study aimed to quantify public acceptability and anticipated uptake with risk stratification incorporated at three points on the bowel cancer screening pathway, compared with current screening in England.

**Methods:**

We conducted an online population-based survey of 1,203 UK adults in 2024. The main outcome measures were overall acceptability of three strategies where risk stratification is introduced at 1) the point of eligibility, 2) the referral threshold and 3) the screening interval, compared with the current programme (screening as usual), and the acceptability of high- and low-risk screening practices at each point. Other outcomes included anticipated uptake of screening and the acceptability of data collection methods.

**Results:**

Acceptability of risk-stratified eligibility and referral thresholds was significantly greater than for screening as usual (*p* < 0.001). There was no difference for stratified intervals. At all three points, more intense screening for those at high-risk was more acceptable and less intense screening for those at low-risk less acceptable when compared with screening as usual (*p* < 0.0001). The anticipated likelihood of taking up screening was also significantly higher if at high-risk and significantly lower if at low-risk, compared with screening as usual for all strategies (*p* < 0.0001).

**Conclusions:**

Participants expressed strong acceptability for risk-stratified bowel cancer screening, particularly for risk-stratified eligibility and thresholds for referral. However, acceptability for less intense screening for those at low-risk was lower than for the current programme and may negatively impact uptake. This suggests that the design and framing of risk groups requires careful consideration and communication with the public.

**Patient and public contribution:**

Three members of the public contributed through online correspondence to the development of the survey and participant-facing documents.

## Highlights

• Risk-stratified bowel screening is generally acceptable to the public.
• Increased screening for high-risk groups is more acceptable than current screening.
• Decreased screening for low-risk groups is less acceptable than current screening.
• Reduced uptake among low risk could impact screening programme outcomes.
• General practice records, genetic and lifestyle data are all acceptable for risk assessment.

## 1 Background

Risk stratification of cancer screening programmes has the potential to prioritise individuals who have the most to gain from screening, thereby improving the distribution of associated harms and benefits (Ghanouni et al., 2020, Hull et al., 2020). This is particularly salient for bowel cancer screening where limited colonoscopy resources introduce additional challenges (Hull et al., 2020). The English Bowel Cancer Screening Programme currently invites males and females aged 56–74 to complete faecal immunochemical test (FIT) screening biennially, with the eligibility criteria currently being expanded to include those aged 50–55. Individuals with a faecal haemoglobin concentration (FHb) above the FIT threshold (currently 120 µg/g for all) are referred for further investigation, typically colonoscopy.

Risk stratification is possible at numerous points on the bowel screening pathway. These include, firstly, varying eligibility criteria according to risk, whereby those considered higher risk are invited to begin screening at an earlier age compared with individuals who are at lower risk. Secondly, risk stratification could be used in combination with absolute FHb to tailor the threshold for referral to colonoscopy according to risk. In this instance, people at higher risk may be referred with a lower FHb. Thirdly, the screening interval, i.e. the time between successive invitations to screening, could be altered, with longer intervals for low-risk groups and shorter intervals for those at higher risk.

At each point on the pathway, establishing a risk-based screening programme will likely require additional data relating to individual risk of bowel cancer to be used within a risk algorithm (Lansdorp-Vogelaar et al., 2022). The optimum combination of included risk factors and the methods by which they are collected remains to be decided (Hull et al., 2020). Data that may be incorporated include age, sex, FIT result(s), lifestyle information and/or genetic data, collected via primary care records, questionnaires and/or saliva samples. Whilst the choice of data will be informed by risk prediction model performance and feasibility, the approach must also be perceived positively by the public, particularly if they are to be actively involved in data collection (Dobrow et al., 2018, Sidani et al., 2009, Bowel cancer - UK NSC).

Risk-stratified cancer screening has largely been met with optimism from the public (Taylor et al., 2023b). In the case of bowel cancer, previous studies have reported strong public acceptability and demonstrated that the public have the potential to prefer such an approach over current age-based screening programme (Dennison et al., 2023, Taylor et al., 2023a). However, quantitative data on public attitudes towards different programme strategies, data collection methods and the potential impact on screening uptake for bowel cancer is lacking. Understanding of these factors is essential to inform the design of future studies and interventions within the bowel screening programme, particularly from the perspective of framing risk groups and public communication.

## 2 Methods

### 2.1 Study design

We conducted an online population-based survey (Appendix 1). Ethical approval was obtained from the University of Cambridge Humanities and Social Sciences Research Ethics Committee (Ref: 23.349). The questions were developed by the study team based on previous research (Dennison et al., 2022, Taylor et al., 2023a). The survey was then piloted with three patient and public involvement (PPI) representatives and refined based on their feedback. The survey included demographic questions to collect data on a range of characteristics, including age, sex, ethnicity, education and socioeconomic status (SES), as well as information on lifestyle, family and personal history of cancer and cancer-related beliefs. Validated measures were used where possible.

To explore attitudes towards risk-stratified bowel cancer screening strategies, participants were firstly provided with background information relating to bowel cancer, including symptoms, the screening process, benefits and harms and risk factors. They were then introduced to four potential screening strategies:

1. Screening as usual: age-based eligibility, fixed FIT threshold and uniform screening interval.
2. Risk-stratified eligibility criteria: screening starting age is dependent on individual risk.
3. Risk-stratified FIT thresholds: threshold for referral to further investigations (typically colonoscopy) is dependent on individual risk.
4. Risk-stratified intervals: interval between FIT screening rounds is dependent on individual risk.

This information was based on the NHS bowel cancer screening leaflet “NHS Bowel Cancer Screening: Helping You Decide” (GOV.UK, 2024), developed using think aloud interviews and tested in a ‘true or false’ format questionnaire (reported separately).

Participants received information about all four strategies prior to answering any survey questions to eliminate potential order effects. They were first asked to consider acceptability of the current bowel cancer screening programme (screening as usual) and whether they would complete FIT screening if offered as part of this programme. Following that, they were briefly reminded of each risk-based strategy before being asked about the acceptability of each programme, the acceptability of four modalities of collecting risk information, anticipated FIT uptake for low, average and high personal risk, and to what extent the risk score would impact their decision to attend a colonoscopy. The four strategies for collecting personalised risk information were:

1. Data already available within general practice (GP) records.
2. Phenotypic/lifestyle data collected via questionnaire.
3. Genetic data obtained via a saliva sample.
4. Strategies 1–3, combined.

Finally, we asked participants how willing they would be to repeat the FIT test if their result was close to the threshold for referral, and about comparative acceptability across the four screening strategies presented to them. Responses were recorded on a five-point Likert scale from ‘extremely acceptable/likely/willing’ to ‘extremely unacceptable/unlikely/unwilling’.

### 2.2 Participants and recruitment

We aimed to recruit a pragmatic sample of 1,200 adult individuals resident in the UK and representative of the UK population in terms of age, sex and ethnicity via the Prolific platform (www.prolific.ac). This is the maximum number of participants for which the Prolific platform could deliver a representative sample at the time of the study. Prolific is an online recruitment platform for researchers where participants volunteer to take part in the study and receive compensation at an agreed hourly rate of £7.50. Following standard recruitment through Prolific, interested participants saw a brief summary of the study before deciding if they wished to take part. If so, they opened the survey and could download PDF copies of the full participant information sheet and consent form.

Written online consent was obtained from each participant. All participants were able to return their submission via the Prolific website at any time, withdrawing consent. Data from returned submissions were not saved or used within the study. We retained data from partial submissions where 95 % of the questions or more were completed if the submission was not returned to Prolific.

### 2.3 Analysis

We used descriptive statistics to characterise participant demographics and beliefs about cancer and screening, as well as attitudes towards the different strategies and intentions to take up FIT screening. For each scenario, we report the number and percentage of participants who found each method of data collection and the screening strategy to be acceptable, and how likely they would be to participate in FIT within each screening programme, according to three hypothetical levels of risk. The Friedman test was used to test for differences across the four scenarios, followed by pairwise comparisons using the Wilcoxon Signed Rank test where the result of the Friedman test was statistically significant.

Logistic regression was used to examine the influence of different demographic characteristics on the likelihood of finding each screening strategy to be ‘somewhat or extremely acceptable’. Additionally, we used logistic regression to examine the odds of finding screening as usual to be acceptable but finding one or more risk-stratified strategy to be unacceptable, adjusting for demographic characteristics. In both cases, we included binary age (younger versus older than 50 years), sex (male versus female), smoking status (non-smoker versus ever smoked), ethnicity (white versus non-white), education (degree versus non-degree), SES (deciles 1–3 versus deciles 4–10) and personal history of cancer (history of cancer versus no history of cancer) in the regression. We report the results of regression analyses as odds ratios (OR) with 95 % confidence intervals (CI).

All analyses were performed using Stata version 17 (StataCorp LLC) with statistical significance set at *p* < 0.01.

## 3 Results

At the time the survey was conducted in January 2024 there were 37,588 participants registered with Prolific who met the eligibility criteria. A total of 1,203 participants completed the survey with an average response time of 15 min 54 s. 123 individuals returned their submission and 16 timed-out. One participant was excluded as they had failed to provide any survey data but had neglected to return their submission. The demographic characteristics of the participants are summarised in Table 1. Reflecting the demographic distribution of the UK population, there was a fairly equal distribution of male and female participants (48.1 % and 51.9 % respectively). The majority of participants were of White ethnicity (86.9 %) and most individuals self-reported as being in the central four socioeconomic deciles (79.7 %). 479 individuals (39.9 %) were educated to degree level and a further 221 (18.4 %) had completed a postgraduate degree. Personal history of cancer was reported by 64 participants (5.3 %) and seven of these (0.6 %) reported a history of bowel cancer specifically. Opinions of bowel cancer screening were positive, with the majority of participants (89.7 %) agreeing with the statement that bowel cancer screening is ‘always a good idea’ and over 90 % of previously invited participants had attended some form of screening.

**Table 1:** Demographic characteristics of participants included in a survey study of an adult sample representative of the UK public in 2024.

**Characteristics**	**Total (%)**
Total N	1203 (100)
Age (years)	
18–29	241 (20.0)
30–39	220 (18.3)
40–49	202 (16.8)
50–59	243 (20.2)
60–69	234 (19.5)
70+	63 (5.2)
Sex	
Male	578 (48.1)
Female	625 (51.9)
Ethnicity	
Asian/Asian British	89 (7.4)
Black, African, Caribbean/Black British	40 (3.3)
Mixed/multiple ethnic group	19 (1.6)
White	1,045 (86.9)
Other	10 (0.8)
Self-reported socioeconomic status	
1–3 (lowest deciles)	145 (12.1)
4–5	400 (33.3)
6–7	558 (46.4)
8–10 (highest deciles)	100 (8.3)
Education level	
Not completed A levels, further education or equivalent	181 (15.0)
Complete A levels, further education or equivalent	320 (26.6)
Completed a bachelor’s degree	479 (39.9)
Completed a postgraduate degree	221 (18.4)
Other	2 (0.2)
Employment status	
Employed full-time	558 (46.4)
Employed part-time	173 (14.4)
Self-employed	105 (8.7)
Studying	36 (3.0)
Not currently working (unemployed, homemaker, retired, disabled, ill)	320 (26.6)
Other	11 (0.9)
Self-reported health status	
Excellent	112 (9.3)
Very good	451 (37.5)
Good	428 (35.6)
Fair	172 (14.3)
Poor	40 (3.3)
Tobacco smoking status	
Never smoked	737 (61.2)
Used to smoke	376 (31.3)
Currently smoke	90 (7.5)
Self-reported weight	
Underweight	32 (2.7)
About right	531 (44.1)
Overweight	640 (53.2)
Personal history of cancer	
Yes	64 (5.3)
No	1,139 (94.7)
Personal history of bowel cancer	
Yes	7 (0.6)
No	1,196 (99.4)
Screening history	
Abdominal aortic aneurysm (ultrasound, men > age 65)	
Invited	55 (4.6)
Attended	57 (103.6)
Bowel (stool sample, all aged 50 or 60–74)	
Invited	366 (30.4)
Attended	340 (92.9)
Breast (mammogram, women aged 50–71)	
Invited	274 (22.8)
Attended	253 (92.3)
Cervical (smear test, women ages 25–64)	
Invited	546 (45.4)
Attended	503 (92.1)
Opinion of bowel cancer screening	
Always a good idea	1,079 (89.7)
Sometimes a good idea/it depends	121 (10.1)
Never a good idea	3 (0.3)

General cancer beliefs are summarised in Supplementary Table S1. Few participants (3.7 %) expressed cancer fatalism (question 3) and the vast majority anticipated survival benefits from early detection (96.3 %; question 5).

### 3.1 Overall acceptability of using different strategies to stratify bowel cancer screening

**Fig. 1:**
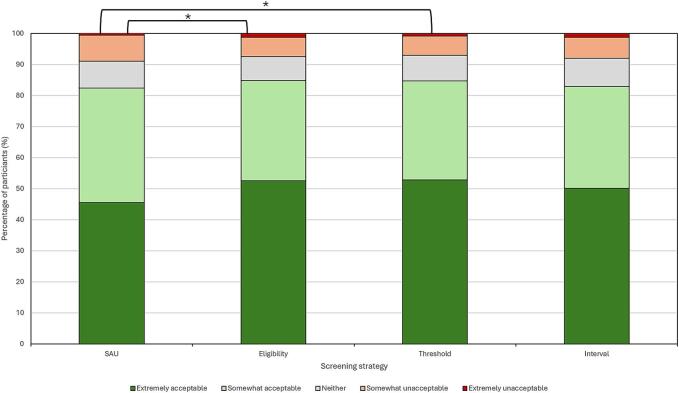
Overall acceptability of risk-stratified screening strategies compared with screening as usual for an adult sample representative of the UK public in 2024. *Wilcoxon Signed Rank test *p* < 0.0001. All other pairwise *p*-values non-significant (*p* > 0.01). *SAU – screening as usual.*

The overall acceptability of each screening strategy is illustrated in Fig. 1 and summarised in Supplementary Table S2. The acceptability of risk-stratification was high, with ≥ 1000 (83.1 %) participants ranking all three strategies as ‘somewhat or extremely’ acceptable. There was a significant difference across the four groups (*p* < 0.0001). In pairwise comparisons, the acceptability of risk-based eligibility criteria and FIT thresholds was significantly higher than for screening as usual (*p* < 0.001). Although, the difference between the acceptability distributions of screening as usual and a programme using risk-stratified screening intervals was not statistically significant (*p* = 0.0454).

**Fig. 2:**
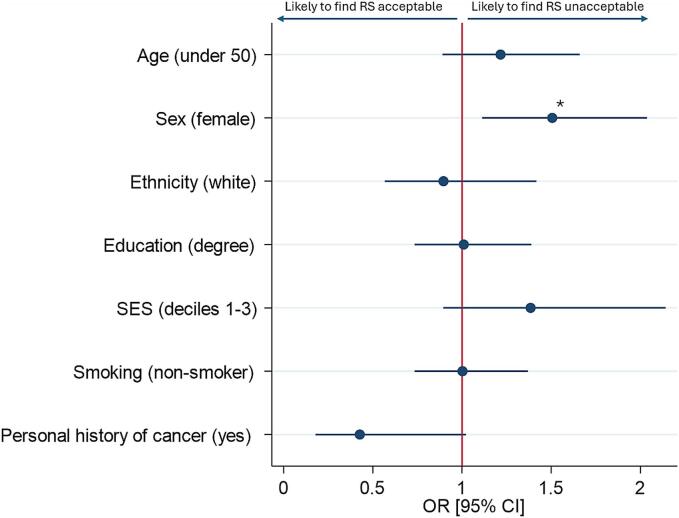
The impact of individual demographics on the odds that participants would find one or more risk-stratified strategy to be unacceptable, after finding screening as usual to be acceptable for an adult sample representative of the UK public in 2024. *p = 0.008. n = 280. *CI – confidence interval; OR – odds ratio; SES – socioeconomic status; RS – risk stratification.*

Female participants were significantly less likely to find risk-stratified screening intervals acceptable than male participants (OR 0.6 [0.5–0.9], *p* = 0.004) (Supplementary Table S3). The difference between female and male participants was not significant for risk-based eligibility and thresholds (*p* = 0.028 and 0.078 respectively). These findings were consistent when examining the impact of demographics on the odds of finding one or more risk-stratified screening strategy unacceptable while considering that age-based screening was acceptable (280 participants). Amongst those, female participants were again significantly more likely to find risk stratification to be unacceptable (OR 1.5 [95 % CI 1.1–2.0], *p* = 0.008) (Fig. 2). No other demographic factors had a significant impact.

### 3.2 Acceptability of high- and low-risk screening practices

**Fig. 3:**
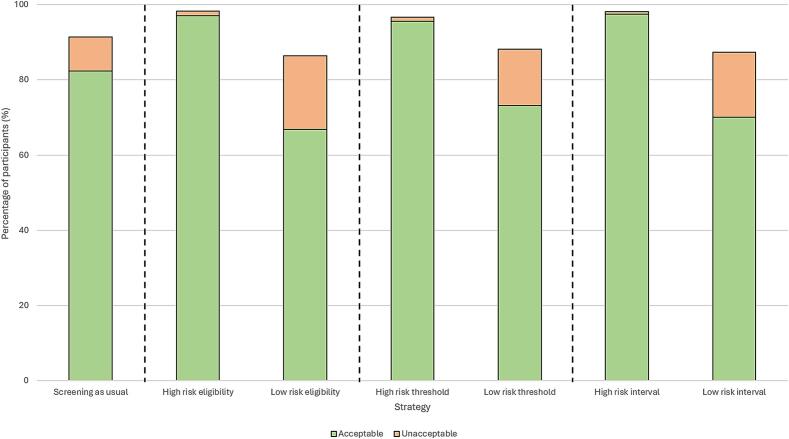
Percentage of participants considering high- and low-risk screening practices ‘somewhat or extremely’ acceptable/unacceptable across screening strategies for an adult sample representative of the UK public in 2024. *SAU – screening as usual.*

The acceptability of differing screening intensity according to risk for each risk-stratified programme compared with screening as usual is illustrated in Fig. 3 and summarised in Supplementary Table S4. Acceptability of high-risk screening practices as part of a programme with risk-based eligibility, FIT thresholds or intervals was significantly higher than for screening as usual at 97.1 %, 95.6 % and 97.5 % respectively (*p* < 0.0001).The majority of participants also reported low-risk screening practices to be ‘somewhat or extremely acceptable’ (66.8 % for eligibility, 73.2 % for threshold and 70.1 % for intervals) and ≤ 3 % of participants considered the strategy ‘extremely unacceptable’. Nevertheless, acceptability of reduced screening for low-risk cohorts was significantly less acceptable than screening as usual across all strategies (*p* < 0.0001). Fewer than 1 % of participants found intensified screening to be unacceptable whereas 15–19.6 % of participants found reducing screening to be unacceptable.

### 3.3 Likelihood of participating in FIT screening according to risk level

**Fig. 4:**
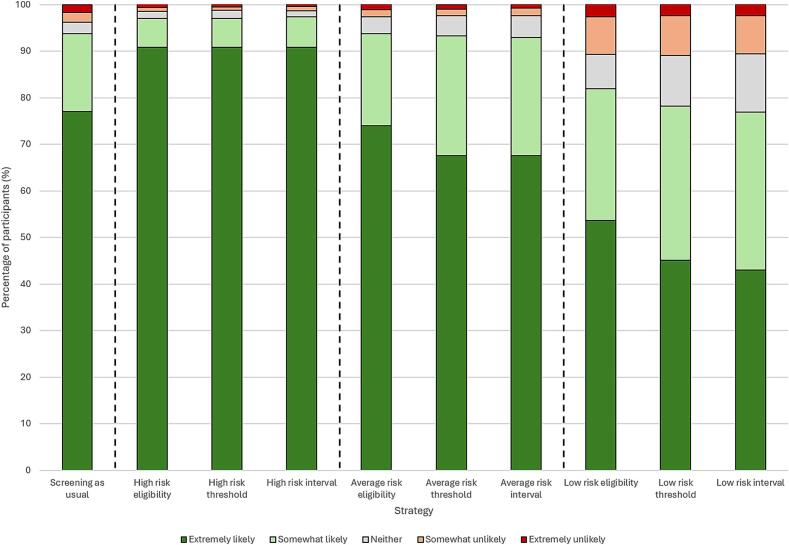
Likelihood of taking up FIT screening at three different risk levels across risk-stratified screening strategies compared with screening as usual for an adult sample representative of the UK public in 2024.

The likelihood of taking up FIT according to hypothetical personal risk is illustrated in Fig. 4 and summarised in Supplementary Table S5. The likelihood of taking up FIT screening differed significantly across the four screening strategies when participants were asked to imagine that they had low, average and high risk levels (*p* < 0.0001). Pairwise comparisons between each risk-based strategy and screening as usual were also significant (*p* < 0.0001), with the exception of risk-stratified eligibility for average-risk (*p* = 0.1544). Consistent with the findings on acceptability, the anticipated likelihood of participants taking up FIT when considering themselves as high risk was greater across all three risk-based strategies (eligibility: 90.9 % extremely likely, 6.2 % somewhat likely; threshold: 90.9 % extremely likely, 6.2 % somewhat likely; interval: 90.9 % extremely likely, 6.5 % somewhat likely; *p* < 0.0001), and lower for all three when considering a hypothetical low risk (eligibility: 53.7 % extremely likely, 28.3 % somewhat likely; threshold: 45.1 % extremely likely, 33.1 % somewhat likely; interval: 43.0 % extremely likely, 34.0 % somewhat likely; *p* < 0.0001). When asked to consider themselves at average risk, there was no significant difference from screening as usual for risk-stratified eligibility, but a small decrease in the likelihood of taking up FIT screening for stratified threshold and interval (*p* < 0.0001).

When asked if they would be willing to undergo repeat FIT testing as part of a screening programme with risk-stratified thresholds for referral to colonoscopy, almost all participants (n = 1,160, 96.4 %) stated that they would be ‘somewhat or extremely willing’ to do so (Supplementary Table S6).

### 3.4 Impact of risk on the decision to attend colonoscopy

The impact of risk on the decision to attend colonoscopy is summarised in Supplementary Table S7 and Supplementary Figure F1. For all three risk-based strategies, participants indicated they would be significantly more likely to attend colonoscopy if they were told they were at high risk of developing bowel cancer than if they were told they were at average or low risk (*p* < 0.0001). Similarly, they reported that they would be significantly more likely to attend if found to be at average risk than if they were at low risk (*p* < 0.0001). For hypothetical average or low risk levels, participants were significantly less likely to attend colonoscopy as part of a programme with risk-stratified eligibility than one with risk-stratified threshold or intervals (*p* < 0.0001).

### 3.5 Acceptability of data collection methods

The acceptability of data collection by GP records, phenotypic/lifestyle data, genetic data or all three as part of each of the three risk-stratified screening strategies is summarised in Supplementary Table S8 and Supplementary Figure F2. Overall acceptability of all four methods was high with > 90 % participants considering each to be ‘somewhat or extremely acceptable’ and only a minority of participants ranking any of the options as ‘somewhat or extremely unacceptable’. The distribution of responses across the four methods was similar for the three risk-based programmes, with the combined approach of GP records, phenotypic/lifestyle and genetic data ranked as most acceptable for all three (*p* > 0.001).

## 4 Discussion

This is the first study to quantify and compare anticipated acceptability and uptake with risk stratification at three points on the bowel cancer screening pathway. In general, acceptability of risk-stratified eligibility criteria, referral thresholds and screening intervals for bowel cancer screening was high. Indeed, participants in this study found risk-based eligibility criteria and thresholds more acceptable than the current age-based approach to screening. This finding is consistent with previous literature documenting public acceptability of risk-stratified cancer screening in general and for bowel cancer specifically (Dennison et al., 2022, Taylor et al., 2023a, Taylor et al., 2023b). We also found that the intensification of screening among high-risk groups was very acceptable, with over 95 % of participants finding this to be ‘somewhat or extremely acceptable’ across all risk-based strategies. Conversely, although over two-thirds of participants reported that low-risk screening practices were ‘somewhat or extremely acceptable’ as part of a risk-stratified programme, reducing screening for low-risk individuals was considered less acceptable than screening as usual by up to 15.6 % of participants and less acceptable than increased screening by up to 30.3 %. Previous literature has similarly reported that increasing screening is more acceptable to the public than a reduction or cessation in screening for those at lower risk (Taylor et al., 2023a, Taylor et al., 2023b), albeit without quantifying these differences or comparing between stratification strategies for bowel cancer screening, as we have done here.

We also tested whether acceptability differed amongst population sub-groups. We found that females may be less likely to accept risk-stratified screening despite finding current screening practices acceptable. A recent report on receptiveness to risk-based innovations similarly found that females were less likely than males to accept screening based on the results of a risk assessment, in combination with other factors (Dennison et al., 2024). This may be attributable to differences in prior screening exposure and experiences of regularly attending screening leading to an endowment effect, as females are invited to breast and cervical screening from a younger age (Kelley-Jones et al., 2021). Reassuringly, there were no differences by ethnicity or SES as uptake typically differs by these groups and it is essential that screening programme changes do not exacerbate inequalities (Hirst et al., 2018).

An important finding is that participants were more likely to report intention to take up FIT screening if they had a hypothetical high risk of developing bowel cancer compared to a hypothetical average or low risk. This suggests that receiving a high-risk estimate may encourage uptake, successfully targeting those who stand to benefit most from screening. Conversely, being labelled as low risk may negatively impact uptake with the proportion of participants indicating an intention to uptake as much as 16.8 % lower than for the current screening programme. As with reduced acceptability of lower intensity screening, this finding confirms previous research suggesting that uptake may consequently decrease by a significant percentage (Evans et al., 2016, Usher-Smith et al., 2021). Sufficient uptake is essential if bowel cancer screening programmes are to achieve a meaningful reduction in incidence and mortality, and to strike an acceptable balance between benefits and harms. A minimum uptake of 52 % is required for the English bowel cancer screening programme to remain viable (Bowel Cancer Screening Annual Report 2021 to 2022). A 17 % reduction in uptake, as reported here, could reduce uptake from current levels of around 69 % to close to this figure, having a significant impact on programme outcomes (Bowel Cancer Screening Annual Report 2021 to 2022). It is possible that increased uptake among high-risk members of the population would go some way towards balancing this effect out. However, prospective uptake at high risk was a maximum of 3.6 % greater than for screening as usual among survey participants, indicating that low-risk estimates have greater potential to impact participation. In this case, for every 5 % of the population classified as low risk, 23 % of the population would need to be classified as high risk in order to balance out uptake. There is also the potential for reduced screening to appear contradictory to traditional screening messaging designed to encourage uptake and promote the value of screening programmes (Taylor et al., 2023c). To avoid this, the framing and communication of low-risk estimates must be carefully considered.

If resources were available, the optimal strategy would be to increase screening amongst those at higher risk without changing screening for average or low risk individuals. However, in the context of limited resources, this is unlikely to be feasible and an alternative approach could be to avoid using the term ‘low risk’. This could be achieved, for example, by having two groups, one labelled average risk and one as higher risk. Screening could be reduced for the average risk group through introduction of a higher FIT threshold or extended screening interval as standard, while categorising a larger proportion as higher risk and offering them intensified screening. However, any change to the screening programme must be communicated with caution to avoid public backlash as was previously observed following introduction of extended cervical cancer intervals for women across Wales. The change was met with low public acceptability (Mulcahy Symmons et al., 2021, Marlow et al., 2022, Nemec et al., 2022) and women felt that inadequately communicated changes left them with unanswered questions and concerns that the resulting screening programme fell short of their expectations (Mulcahy Symmons et al., 2021).

Finally, we found that the acceptability of data collection via GP records, lifestyle surveys and cheek swabs to obtain genetic information was high, with over 90 % of participants rating all data collection strategies to be acceptable and the most acceptable option being a combination of all three methods.

### 4.1 Strengths and limitations

A key strength of this study is the use of information text developed with public input through a series of think aloud interviews and evaluated for comprehension using a user testing survey. Similarly, input from three PPI representatives ensured the readability of the resulting text. Additionally, all information relating to the screening strategies was delivered to participants ahead of any survey questions to limit order effects. Finally, the survey design accommodated collection of information on a wide range of variables, allowing investigation of acceptability, anticipated uptake and data collection.

The uptake rates reported here are measures of intention and are higher than rates of uptake observed in practice. Due to the recognised intention-action gap, this difference makes it challenging to translate the findings to expected uptake rates in practice. Additionally, we cannot comment on how far self-perceived risk of bowel cancer may have impacted survey responses, particularly where participants were asked to consider having different risk levels. Furthermore, the use of general risk groupings instead of providing detailed quantitative risk information may have influenced participant views on acceptability or their decisions to take up screening. Finally, as well as the potential for self-selection bias, the survey participants were well educated and such individuals may be more likely to attend and adhere to screening than the population as a whole (Damiani et al., 2015, Power et al., 2009).

## 6 Conclusion

The results of this online survey study demonstrate that introducing risk stratification at three distinct points on the bowel cancer screening pathway is largely acceptable to the public. However, our findings also highlight that decreased intensity screening is less acceptable than increased screening and uptake may be significantly lower among those labelled as low risk. These attitudes must be considered when designing risk-stratified bowel cancer screening programmes, specifically the selection of risk groups and framing for those at lower risk.

## Ethical approval and consent

Ethical approval was obtained from the University of Cambridge Humanities and Social Sciences Research Ethics Committee (Ref: 23.349). All participants gave consent before completing the survey. The study was performed in accordance with the Declaration of Helsinki.

## CRediT authorship contribution statement

**Lily C. Taylor:** Conceptualization, Data curation, Formal analysis, Investigation, Methodology, Project administration, Visualization, Writing – original draft, Writing – review & editing. **Rebecca A. Dennison:** Writing – review & editing, Supervision, Formal analysis, Conceptualization. **Juliet A. Usher-Smith:** Writing – review & editing, Supervision, Methodology, Funding acquisition, Conceptualization.

## Declaration of competing interest

The authors declare that they have no known competing financial interests or personal relationships that could have appeared to influence the work reported in this paper.

## Data Availability

Data are available via the University of Cambridge Data Repository (https://doi.org/10.17863/CAM.111776). The study protocol, participant information sheet and consent form will be available on the repository. Data will be available upon publication with no end date as indicated.
