# Supplementary material for: Public acceptability and anticipated uptake of risk-stratified bowel cancer screening in the UK: An online survey
Source: Prev Med Rep. 2024 Nov 10;48:102927. doi: 10.1016/j.pmedr.2024.102927 (PMC11614824; doi:10.1016/j.pmedr.2024.102927)
Supplement: Supplementary Data 1 [file mmc1.pdf]

Public acceptability and anticipated uptake of risk-stratified bowel cancer screening in the UK: an online survey

Supplementary information

Supplementary Table S.1 General cancer beliefs of participants included in a survey study of an adult sample representative of the UK public in 2024

	Strongly agree/agree (n/%)	Neither agree nor disagree (n/%)	Disagree or strongly disagree (n/%)
1. "These days, many people with cancer can expect to continue with normal activities and responsibilities."	828 (68.8)	253 (21.1)	121 (10.1)
2. "Most cancer treatment is worse than the cancer itself."	303 (25.2)	495 (41.2)	404 (33.6)
3. "I would NOT want to know if I have cancer."	44 (3.7)	112 (9.3)	1,046 (87.0)
4. "Cancer can often be cured."	811 (67.5)	304 (25.3)	87 (7.2)
5. "Going to the doctor as quickly as possible after noticing a symptom of cancer could increase the chances of surviving."	1,157 (96.3)	35 (2.9)	10 (0.8)
6. "Some people think a diagnosis of cancer is a death sentence."	1,044 (86.9)	109 (9.1)	49 (4.1)

Supplementary Table S.2 Overall acceptability of risk-stratified screening strategies compared with screening as usual for an adult sample representative of the UK public in 2024

**Acceptability**	**Screening as usual (n/%)**	**Risk-stratified eligibility (n/%)**	**Risk-stratified threshold (n/%)**	**Risk-stratified interval (n/%)**
Friedman test *p*-value	<0.0001			
Wilcoxon sign rank *p*-value	Ref	0.0003	0.0001	0.0454
Median (Inter-quartile range)	Somewhat acceptable (Extremely acceptable-somewhat acceptable)	Somewhat acceptable (Extremely acceptable-somewhat acceptable)	Somewhat acceptable (Extremely acceptable-somewhat acceptable)	Somewhat acceptable (Extremely acceptable-somewhat acceptable)
Extremely acceptable	547 (45.5)	632 (52.5)	635 (52.8)	604 (50.2)
Somewhat acceptable	444 (36.9)	389 (32.3)	384 (31.9)	395 (32.8)
Neither	104 (8.7)	92 (7.7)	98 (8.2)	108 (9.0)
Somewhat unacceptable	100 (8.3)	75 (6.2)	76 (6.3)	80 (6.7)
Extremely unacceptable	8 (0.7)	15 (1.3)	10 (0.8)	16 (1.3)

Supplementary Table S.3 The impact of individual demographics on the likelihood that participants would find each risk-stratified screening strategy acceptable for an adult sample representative of the UK public in 2024

**Demographic characteristics**	**OR [95% CI]**	***p*-value**
	Eligibility	
Age (under 50)	1.0 [0.7-1.4]	0.982
Sex (female)	0.7 [0.5-1.0]	0.028
Ethnicity (white)	1.1 [0.7-1.8]	0.598
Education (degree)	1.2 [0.8-1.7]	0.334
SES (lower)	0.8 [0.5-1.3]	0.454
Smoking (non-smoker)	0.9 [0.7-1.3]	0.707
Personal cancer history (yes)	2.2 [0.9-5.6]	0.099
	Threshold	
Age (under 50)	1.0 [0.7-1.3]	0.814
Sex (female)	0.8 [0.5-1.0]	0.078
Ethnicity (white)	1.3 [0.8-2.0]	0.313
Education (degree)	1.2 [0.9-1.7]	0.304
SES (lower)	0.7 [0.5-1.2]	0.180
Smoking (non-smoker)	1.1 [0.8-1.5]	0.751
Personal cancer history (yes)	2.8 [1.0-7.8]	0.054
	Interval	
Age (under 50)	1.0 [0.7-1.3]	0.897
Sex (female)	0.6 [0.5-0.9]	**0.004**
Ethnicity (white)	1.0 [0.6-1.6]	0.939
Education (degree)	1.3 [0.9-1.8]	0.109
SES (lower)	0.9 [0.6-1.4]	0.545
Smoking (non-smoker)	0.9 [0.7-1.3]	0.696
Personal cancer history (yes)	2.6 [1.0-6.6]	0.047

Supplementary Table S.4 Acceptability of high- and low-risk screening practices according to risk-stratified screening strategy compared with screening as usual for an adult sample representative of the UK public in 2024

Acceptability	Screening as usual (n/%)	Eligibility (n/%)	Threshold (n/%)	Interval (n/%)
High-risk screening practices				
Friedman test *p*-value	<0.0001			
Wilcoxon sign rank *p*-value	REF	<0.0001	<0.0001	<0.0001
Median (Inter-quartile range)	Somewhat acceptable (Extremely acceptable-somewhat acceptable)	Extremely acceptable (Extremely acceptable-extremely acceptable)	Extremely acceptable (Extremely acceptable-somewhat acceptable)	Extremely acceptable (Extremely acceptable-extremely acceptable)
Extremely acceptable	547 (45.5)	1,040 (86.5)	884 (73.5)	986 (82.0)
Somewhat acceptable	444 (36.9)	127 (10.6)	266 (22.1)	187 (15.5)
Neither	104 (8.7)	21 (1.8)	40 (3.3)	23 (1.9)
Somewhat unacceptable	100 (8.3)	5 (0.4)	11 (0.9)	5 (0.4)
Extremely unacceptable	8 (0.7)	10 (0.8)	2 (0.2)	2 (0.2)
Low-risk screening practices				
Friedman test *p*-value	<0.0001			
Wilcoxon sign rank *p*-value	REF	<0.0001	<0.0001	<0.0001
Median (Inter-quartile range)	Somewhat acceptable (Extremely acceptable-somewhat acceptable)	Somewhat acceptable (Somewhat acceptable-neither)	Somewhat acceptable (Extremely acceptable-neither)	Somewhat acceptable (Extremely acceptable-neither)
Extremely acceptable	547 (45.5)	267 (22.2)	409 (34.0)	325 (27.0)
Somewhat acceptable	444 (36.9)	536 (44.6)	472 (39.2)	518 (43.1)
Neither	104 (8.7)	163 (13.6)	142 (11.8)	151 (12.6)
Somewhat unacceptable	100 (8.3)	200 (16.6)	149 (12.4)	176 (14.6)
Extremely unacceptable	8 (0.7)	37 (3.1)	31 (2.6)	33 (2.7)

Supplementary Table S.5 The likelihood of taking up FIT screening at different risk levels across risk-stratified screening strategies compared with screening as usual for an adult sample representative of the UK public in 2024

**Likelihood of taking up FIT**	**Screening as usual (n/%)**	**Eligibility (n/%)**	**Threshold (n/%)**	**Interval (n/%)**
High risk				
Friedman test *p*-value	<0.0001			
Wilcoxon sign rank *p*-value	REF	<0.0001	<0.0001	<0.0001
Median (Inter-quartile range)	Extremely acceptable (Extremely acceptable-extremely acceptable)	Extremely acceptable (Extremely acceptable-extremely acceptable)	Extremely acceptable (Extremely acceptable-extremely acceptable)	Extremely acceptable (Extremely acceptable-extremely acceptable)
Extremely likely	927 (77.1)	1,093 (90.9)	1,093 (90.9)	1,093 (90.9)
Somewhat likely	201 (16.7)	74 (6.2)	75 (6.2)	78 (6.5)
Neither	29 (2.4)	18 (1.5)	20 (1.7)	15 (1.3)
Somewhat unlikely	27 (2.2)	9 (0.8)	8 (0.7)	11 (0.9)
Extremely unlikely	19 (1.6)	9 (0.8)	7 (0.6)	6 (0.5)
Average risk				
Friedman test *p*-value	<0.0001			
Wilcoxon sign rank *p*-value	REF	0.1544	<0.0001	<0.0001
Median (Inter-quartile range)	Extremely acceptable (Extremely acceptable-extremely acceptable)	Extremely acceptable (Extremely acceptable-somewhat acceptable)	Extremely acceptable (Extremely acceptable-somewhat acceptable)	Extremely acceptable (Extremely acceptable-somewhat acceptable)
Extremely likely	927 (77.1)	890 (74.0)	813 (67.6)	813 (67.6)
Somewhat likely	201 (16.7)	238 (19.8)	309 (25.7)	306 (25.4)
Neither	29 (2.4)	43 (3.6)	53 (4.4)	55 (4.6)
Somewhat unlikely	27 (2.2)	18 (1.5)	17 (1.4)	20 (1.7)
Extremely unlikely	19 (1.6)	14 (1.2)	11 (0.9)	9 (0.8)
Low risk				
Friedman test *p*-value	<0.0001			
Wilcoxon sign rank *p*-value	REF	<0.0001	<0.0001	<0.0001
Median (Inter-quartile range)	Extremely acceptable (Extremely acceptable-extremely acceptable)	Extremely acceptable (Extremely acceptable-somewhat acceptable)	Somewhat acceptable (Extremely acceptable-somewhat acceptable)	Somewhat acceptable (Extremely acceptable-somewhat acceptable)

Extremely likely	927 (77.1)	646 (53.7)	542 (45.1)	517 (43.0)
Somewhat likely	201 (16.7)	340 (28.3)	398 (33.1)	409 (34.0)
Neither	29 (2.4)	89 (7.4)	131 (10.9)	150 (12.5)
Somewhat unlikely	27 (2.2)	96 (8.0)	103 (8.6)	98 (8.2)
Extremely unlikely	19 (1.6)	32 (2.7)	29 (2.4)	29 (2.4)

Supplementary Table S.6 Willingness to undergo a repeat FIT if found to have a faecal haemoglobin concentration near the threshold for referral to colonoscopy as part of a screening programme with risk-stratified referral thresholds for an adult sample representative of the UK public in 2024

**Willingness to repeat FIT**	**n/%**
Extremely willing	952 (79.1)
Somewhat willing	208 (17.3)
Neither	22 (1.8)
Somewhat unwilling	13 (1.1)
Extremely willing	8 (0.7)

Supplementary Table S.7 The likelihood of personal risk score impacting the decision to attend colonoscopy for an adult sample representative of the UK public in 2024

	**Much more likely**	**A bit more likely**	**No change**	**A bit less likely**	**Much less likely**
High risk					
p-value (Friedman)	<0.0001				
Eligibility	956 (79.5)	121 (10.1)	118 (9.8)	4 (0.3)	4 (0.3)
Threshold	969 (80.6)	103 (8.6)	125 (10.4)	2 (0.2)	4 (0.3)
Interval	978 (81.3)	92 (7.7)	125 (10.4)	3 (0.3)	5 (0.4)
Average risk					
p-value	<0.0001				
Eligibility	480 (39.9)	429 (35.7)	266 (22.1)	23 (1.9)	5 (0.4)
Threshold	543 (45.1)	398 (33.1)	243 (20.2)	14 (1.2)	5 (0.4)
Interval	575 (47.8)	360 (29.9)	246 (20.5)	15 (1.3)	7 (0.6)
Low risk					
p-value	<0.0001				
Eligibility	185 (15.4)	338 (28.1)	424 (35.3)	204 (17.0)	52 (4.3)
Threshold	192 (16.0)	373 (31.0)	422 (35.1)	181 (15.1)	35 (2.9)
Interval	197 (16.4)	394 (32.8)	413 (34.3)	156 (13.0)	43 (3.6)

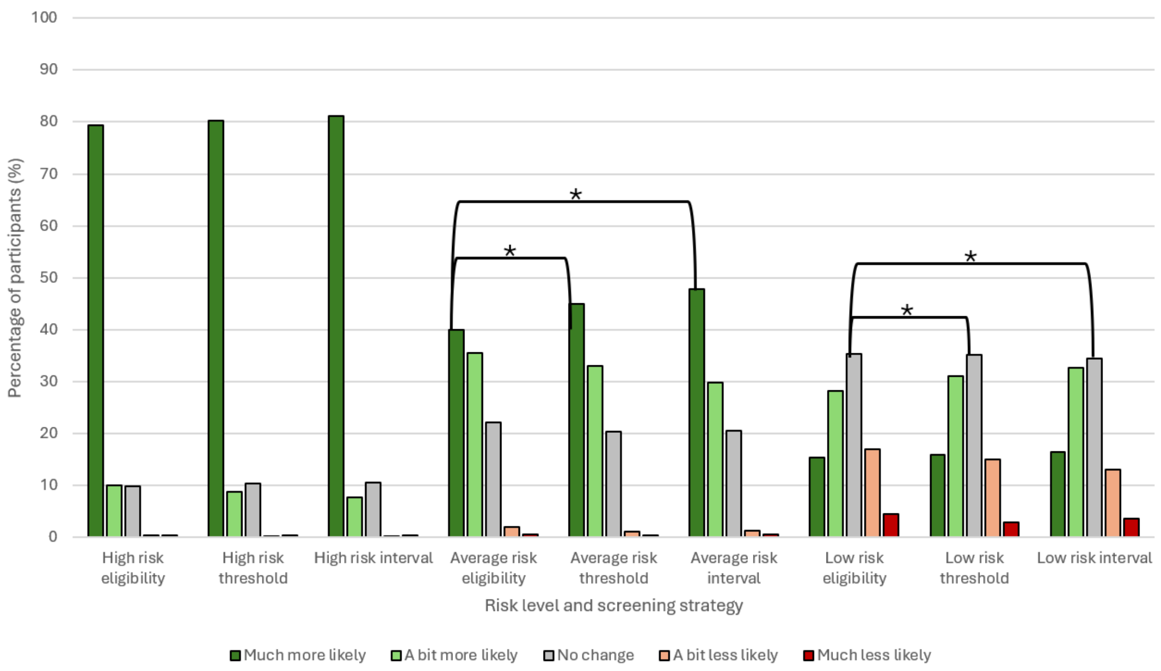

Supplementary Figure F.1 The likelihood that personal risk score would impact the decision to attend colonoscopy according to risk-stratified screening strategy for an adult sample representative of the UK public in 2024

*Wilcoxon Signed Rank test $p<0.0001$. All pairwise comparisons within the same screening strategy were statistically significant ($p<0.0001$).

Supplementary Table S.8 Acceptability of data collection methods summarised by risk-stratified screening strategy for an adult sample representative of the UK public in 2024

	Extremely acceptable (n/%)	Somewhat acceptable (n/%)	Neither acceptable nor unacceptable (n/%)	Somewhat unacceptable (n/%)	Extremely unacceptable (n//%)
Risk-stratified eligibility					
Friedman test *p*-value	<0.0001				
GP records	779 (64.8)	314 (26.1)	54 (4.5)	44 (3.7)	12 (1.0)
Phenotypic data	752 (62.6)	346 (28.8)	63 (5.2)	31 (2.6)	11 (0.9)
Genetic data	796 (66.2)	311 (25.9)	57 (4.7)	28 (2.3)	11 (0.9)
GP records, phenotypic and genetic data	811 (67.4)	313 (26.0)	45 (3.7)	25 (2.1)	9 (0.8)
Risk-stratified threshold					
Friedman test *p*-value	0.0001				
GP records	810 (67.3)	308 (25.6)	41 (3.4)	35 (2.9)	9 (0.8)
Phenotypic data	763 (63.4)	351 (29.2)	53 (4.4)	27 (2.2)	9 (0.8)
Genetic data	812 (67.5)	307 (25.5)	53 (4.4)	23 (1.9)	8 (0.7)
GP records, phenotypic and genetic data	826 (68.7)	297 (24.7)	55 (4.6)	19 (1.6)	6 (0.5)
Risk-stratified interval					
Friedman test *p*-value	0.0007				
GP records	803 (66.8)	317 (26.4)	41 (3.4)	34 (2.8)	8 (0.7)
Phenotypic data	773 (64.3)	335 (27.9)	62 (5.2)	27 (2.2)	6 (0.5)
Genetic data	815 (67.8)	295 (24.5)	62 (5.2)	23 (1.9)	7 (0.6)
GP records, phenotypic and genetic data	826 (68.7)	302 (25.1)	49 (4.1)	22 (1.8)	4 (0.3)

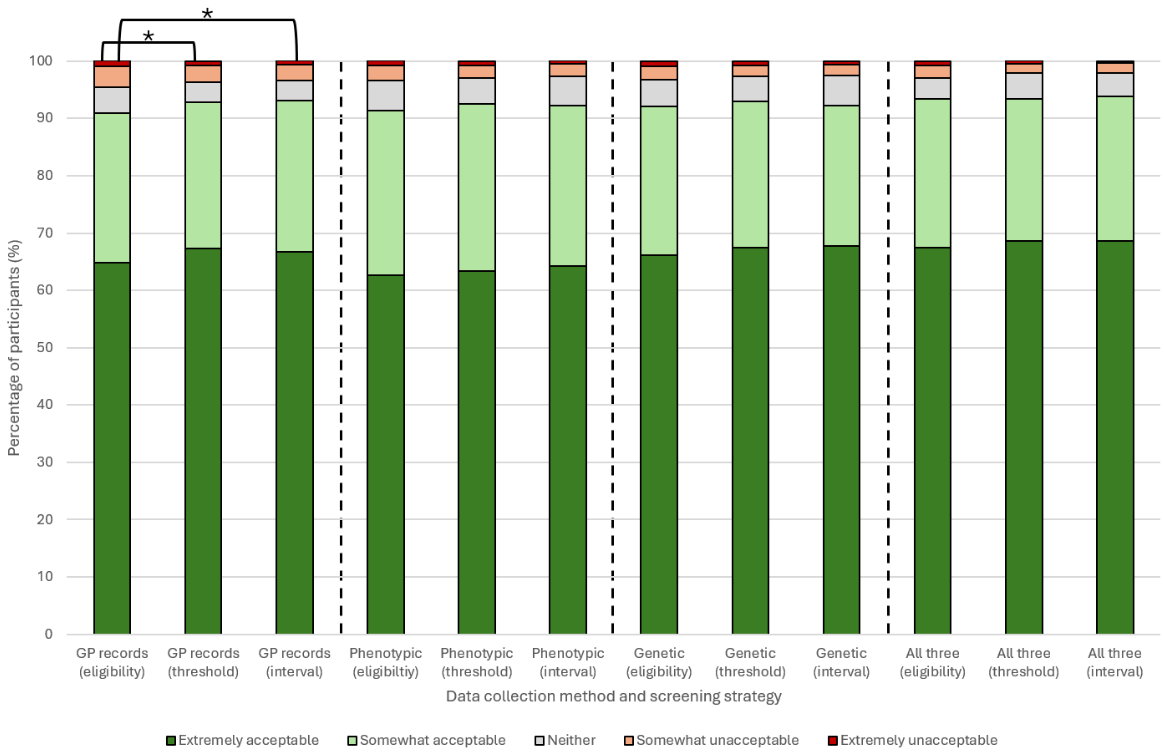

Supplementary Figure F.2 Acceptability of data collection methods illustrated by risk-stratified screening strategy for an adult sample representative of the UK public in 2024

*Wilcoxon Signed Rank test p<0.0001. All other p-values comparing data collection methods *between* strategies were non-significant (p>0.001). All p-values comparing each data collection method *within* the same strategy were significant (p<0.001).
